# Pd(II) Binding Strength of a Novel Ambidentate Dipeptide-Hydroxypyridinonate Ligand: A Solution Equilibrium Study

**DOI:** 10.3390/molecules27144667

**Published:** 2022-07-21

**Authors:** Linda Bíró, András Ozsváth, Réka Kapitány, Péter Buglyó

**Affiliations:** Department of Inorganic & Analytical Chemistry, Faculty of Science and Technology, University of Debrecen, Egyetem tér 1, H-4032 Debrecen, Hungary; linda.biro@science.unideb.hu (L.B.); ozsvath.andras92@gmail.com (A.O.); kapitany.reka95@gmail.com (R.K.)

**Keywords:** multitargeted, palladium, anticancer, speciation, stability constant, ambidentate, hydroxypyridinone

## Abstract

A novel ambidentate dipeptide conjugate (H(L1)) containing N-donor atoms of the peptide part and an (O,O) chelate at the hydroxypyridinone (HP) ring is synthesized and characterized. It is hoped that this chelating ligand can be useful to obtain multitargeted Co(III)/Pt(II) dinuclear complexes with anticancer potential. The Pd(II) (as a Pt(II) model but with faster ligand exchange reactions) binding strength of the ligand was studied in an aqueous solution with the combined use of pH-potentiometry and NMR. In an equimolar solution, (L1)^−^ was found to bind Pd(II) via the terminal amino and increasing number of peptide nitrogens of the peptide backbone over a wide pH range. At a 2:1 Pd(II) to ligand ratio, the presence of [Pd_2_H_–x_(L1)] (x = 1–4) species, with high stability and with the coordination of the (O,O) chelating set of the ligand, was detected. The reaction of H(L1) with [Co(tren)]^3+^ (tren = tris(2-aminoethyl)amine) indicated the exclusive binding of (L1)^−^ via its (O,O) donor atoms to the metal unit, while treatment of the resulting Co-complex with Pd(II) afforded the formation of a Co/Pd heterobimetallic complex in solution with an (NH_2_, N_amide_) coordination of Pd(II). Shortening the peptide backbone in H(L1) by one peptide unit compared to the structurally similar ambidentate chelator consisting of three peptide bonds resulted in the slightly more favorable formation of the N-coordinated Pd(II) species, allowing the tailoring of the coordination properties.

## 1. Introduction

Peptide conjugates incorporating a separated chelating set in addition to the peptide backbone may provide a promising platform to construct bimetallic complexes with targeted anticancer potential [1,2,3]. Besides the (NH_2_,N_amide_) coordinated platinum group metals (PGM; Pt(II), Pd(II) as a Pt(II) model exhibiting, however, faster ligand exchange processes, half-sandwich type organo-Ru(II), -Os(II) or -Rh(III) and -Ir(III)), all with proven anticancer activity, a separated (O,O) chelating unit of the ligand with a distinct biological function (e.g., enzyme inhibition, strong iron sequestering capability to disturb the Fe(III) homeostasis of the fast proliferating cancer cells, etc.) can bind a [Co(4N)]^3+^ (4N = tren, tpa) (tpa = tris(2-pyridylmethyl)amine) unit [4,5,6,7]. Various types of Pd(II) complexes themselves exhibit remarkable anticancer potential sometimes via a different mechanism of action compared to the platinum(II) analogues [8,9,10,11]. Rationally tailored redox properties of the above type Co(III) complexes enable selective reduction in Co(III) to Co(II) under the hypoxic environment of the cancer cells only [12,13,14]. As a consequence, the free (O,O) chelating groups can exert their biological effects after the dissociation of the Co(II) complex having lower thermodynamic stability and faster ligand exchange properties compared to those of the parent complex. Furthermore, Co(II) ion was also shown to exhibit anticancer potential via the changing in the transcription of some genes (e.g., HIF-1α, p53, PCNA, etc.), generating ROS and causing subsequent apoptosis due to mitochondrial DNA damage [15,16].

To construct these types of complexes, it is important to explore the donor atom ((O,O) vs. (N,N)) preferences of the metal building blocks (Co(III) vs. PGMs). Recently we have shown that the [Co(4N)]^3+^ cation exhibits a preference toward the (O,O) chelates (e.g., hydroxamate or hydroxypyridinonate (HP)) during the reaction with similar types of ambidentate chelators. On the contrary, PGMs were found to bind preferably via the (NH_2_,N_amide_) chelating set of these ligands; although with HPs at an excess of metal ion (O,O), coordination was also revealed [4,5,7]. Notably, for peptide hydroxamates Pd(II)-assisted hydrolysis of the hydroxamate group followed by a redox reaction between Pd(II) and the hydroxylammonium ion formed under acidic conditions was detected, resulting in the formation of Pd(0) [7].

Besides the type, basicity, and size of the chelates that can be formed in peptide conjugates, the number of the available amide nitrogens for coordination not only provides structural variations but will determine the distance between the separated metal ion entities and can therefore significantly contribute to the stoichiometry and stability of the Co(III)/PGM bimetallic complexes with likely anticancer potential.

**Scheme 1:**
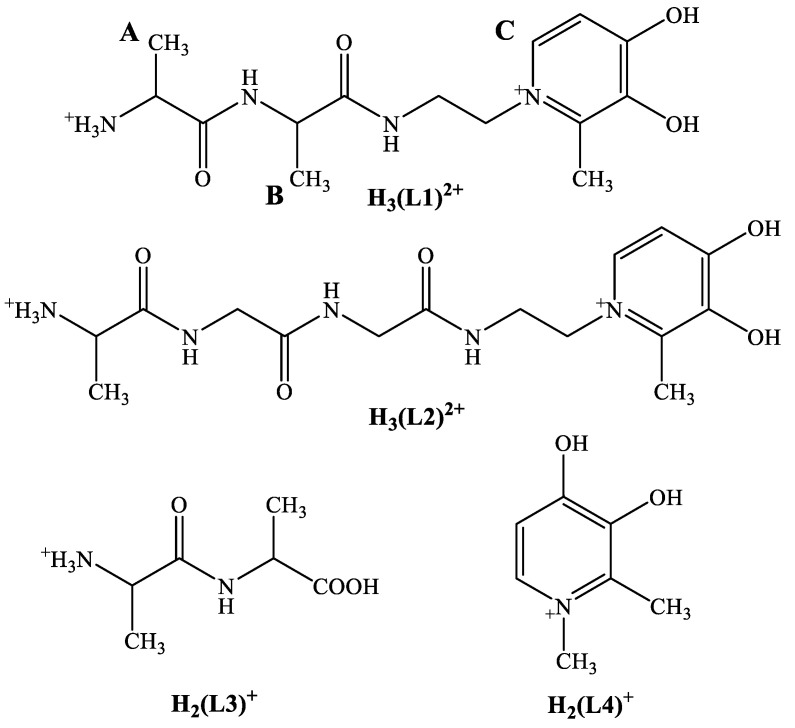
Structural formula of the fully protonated form of the novel ligand, H_3_(L1)^2+^, with the labeling scheme (**A**–**C**) of some protons for the NMR studies together with the formulae of some structural models, H_3_(L2)^2+^–H_2_(L4)^+^.

The aim of the present work, therefore, was the synthesis, characterization of a novel dipeptide-HP conjugate ligand (2-(2-methyl-3-hydroxy-4-pyridinon-1-yl)ethyl)-*N*-l-alanyl-l-alanyl-amide (H(L1), see structure in Scheme 1), and to explore its Pd(II) and [Co(4N)]^3+^ (4N = tren) binding capability in solution with the combined use of pH-potentiometry and NMR techniques. We also report on the donor atom preferences of Pd(II) and [Co(tren)]^3+^ toward H(L1) studied by ^1^H NMR to assist in the development of Co/Pd(Pt) heterobimetallic complexes. Exploring these interactions may give a useful platform for the successful solid-phase synthesis of bimetallic complexes, furthermore, and information about the solution behavior of the administered PGM/Co complex with antiproliferative potential after hypoxia activation.

## 2. Experimental

### 2.1. Materials

THF and methanol were purified according to literature methods [17]. Maltol, benzyl chloride, 1,2-diaminoethane, *N*-methylmorpholine (NMM), and trimethylsilyl propanoic acid (TSP) were purchased from Sigma-Aldrich (Burlington, MA, USA). Z-Ala-Ala-OH was delivered by Bachem, Pd/C (10 %), and EtOCOCl by Merck while MgSO_4_ was obtained from VWR. 3-benzyloxy-2-methyl-4-pyrone and 1-(2-aminoethyl)-3-benzyloxy-2-methyl-4-pyridinone hydrochloride as precursors for the synthesis of H(L1) were prepared according to previously described methods [18,19]. K_2_[PdCl_4_] (Sigma-Aldrich) was used for preparing the Pd(II) stock solutions by dissolving it in doubly deionized and ultrafiltered water from a Milli-Q RG (Millipore) water purification system and by adding a known amount of HCl to avoid hydrolysis. [Co(tren)Cl_2_]Cl was synthesized as previously reported [13].

### 2.2. Syntheses

#### 2.2.1. Synthesis of Benzyl-(1-((1-((2-(3-(Benzyloxy)-2-methyl-4-oxopyridin-1(4H)-yl)ethyl)amino)-1-oxopropan-2-yl)amino)-1-oxopropan-2-yl)carbamate, (**1**)

Briefly, 1.20 g (3.62 mmol) of 1-(2-aminoethyl)-3-benzyloxy-2-methyl-4-pyridinone hydrochloride was added to 420 mg (7.48 mmol) KOH in 20 mL abs. methanol. The solution was stirred for 15 min under an N_2_ atmosphere at 0 °C, then the precipitated KCl was removed by the filtration of this solution into a three-neck flask under nitrogen. In another flask, Z-L-Ala-L-Ala-OH (0.94 g; 3.19 mmol) was dissolved in 50 mL abs. THF at 0 °C and then NMM (394 µL; 3.59 mmol) and EtOCOCl (405 µL; 4.24 mmol) were added. This solution was stirred for 30 min in an ice-bath while N-methylmorpholinium chloride was formed. The mixture was filtered into a dropping funnel under N_2_ and was added dropwise to the former solution. The obtained reaction mixture was stirred at 0 °C for 1 h, and then left overnight at ambient temperature. The solvent was removed under vacuum and then the residue was purified by flash chromatography on a silica gel column (eluent: CHCl_3_: MeOH = 4:1). The product was recrystallized from ethyl acetate. Yield: 0.59 g (34%).

^1^H NMR (400 MHz, DMSO-d_6_ (δ_DMSO-d6_ = 2.50 ppm)) δ (ppm): 8.06 (1H, t, NH); 7.96 (1H, d, NH); 7.34 (11H, m, Ar-H); 6.11 (1H, d, CH); 5.02 (2H, q, CH_2_); 5.00 (2H, s, CH_2_); 4.18 (1H, q, CH); 4.05 (1H, q, CH); 3.92 (2H, m, CH_2_); 3.30 (2H, m, CH_2_); 2.19 (3H, s, CH_3_); 1.18 (3H, d, CH_3_); 1.17 (3H, d, CH_3_). ^13^C NMR (90 MHz, CDCl_3_, 77.16 ppm) δ (ppm): 12.8 (ring-*C*H_3_), 18.5 and 18.6 (2x Ala-*C*H_3_), 38.8 (-*C*H_2_-CH_2_-), 49.6 and 51.3 (2x Ala-*C*H), 53.6 (-CH_2_-*C*H_2_-), 66.8 and 74.0 (-*C*H_2_- of the protecting groups), 115.4 (malt ring *C*H), 127.8, 128.1, 128.6 and 128.9 (ring *C*Hs of the protecting groups), 136.6 and 137.8 (-CH_2_-*C*= of the protecting groups) 141.0 (malt ring-*C*H), 144.3 and 145.4 (malt ring-*C*), 156.3 (*C*=O of the protecting group), 170.8 (*C*=O malt ring), 172.7 and 173.8 (2x Ala *C*=O). Anal. Required for C_29_H_34_N_4_O_6_: C, 65.15, H, 6.41, N, 10.48%. Found: C, 64.86, H, 6.30, N, 10.27%.

#### 2.2.2. Synthesis of the Trifluoroacetate Salt of 2-Amino-*N*-(1-((2-(3-Hydroxy-2-methyl-4-oxopyridin-1(4H)-yl)ethyl)amino)-1-oxopropan-2-yl)propanamide, H(L1) CF_3_COOH

**1** (0.59 g; 1.10 mmol) was dissolved in 20 mL abs. methanol, and then Pd/C (212 mg) and CF_3_COOH (101 µL; 1.33 mmol) were added. After a 4 h stirring in an atmosphere of H_2_ (ca. 2 atm), the catalyst was filtered off and the filtrate was evaporated in a vacuum. The residue was treated with dry diethyl ether to give the solid product as the corresponding trifluoroacetate salt. Yield: 0.42 g (89 %).

^1^H NMR (400 MHz, D_2_O (δ_D2O_ = 4.79 ppm)) δ (ppm): 7.60 (1H, d, CH); 6.57 (1H, d CH); 4.30 (2H, t, CH_2_); 4.23 (1H, q CH); 4.01 (1H, q, CH); 3.61 (2H, m, CH_2_); 2.45 (3H, s, CH_3_); 1.45 (3H, d, CH_3_); 1.25 (3H, d, CH_3_). ^13^C NMR (90 MHz, D_2_O/*C*H_3_OH 49.50 ppm) δ (ppm): 12.6 (ring-*C*H_3_), 17.0 and 17.3 (2x Ala-*C*H_3_), 39.1 (-*C*H_2_-CH_2_-), 50.0 and 50.2 (2x Ala-*C*H), 54.9 (-CH_2_-*C*H_2_-), 112.1 (malt ring *C*H), 139.1 (malt ring-*C*), 139.7 (malt ring-*C*H), 144.4 (malt ring-*C*), 164.5 (*C*=O malt ring), 170.8 and 175.4 (2x Ala *C*=O). Anal. Required for C_16_H_23_F_3_N_4_O_6_: C, 45.28, H, 5.46, N, 13.20%. Found: C, 44.93, H, 5.48, N, 13.11%.

### 2.3. Solution Studies

pH-potentiometric measurements were carried out at 25.0 °C and at a constant ionic strength of 0.20 M (0.10 M KCl + 0.10 M KNO_3_). The presence of chloride ions as competitor ligands ensured to shift the complex formation to the measurable pH range. However, the presence of more than 0.10 M chloride ion may hinder the equilibrium studies due to slow complexation processes [20]. Carbonate-free KOH solution of known concentration (ca. 0.2 M) was used as titrant. The exact concentrations of HCl and KOH solutions were determined by potentiometric titrations using Gran’s method [21]. A Mettler Toledo T5 titrator equipped with a Metrohm 6.0234.100 combined glass electrode was used for titrations. The electrode system was calibrated according to Irving et al. [22] and therefore the pH-metric readings were converted into hydrogen ion concentration. The water ionization constant was 13.76 ± 0.01 under the conditions applied. The initial volume of the samples was 15.00 mL. The ligand concentration was 2.55 mM; 1:1, 1:2, and 2:1 metal ion to ligand ratios were studied and the samples were stirred and completely deoxygenized by bubbling with purified argon for ca. 15 min before measurements. The titrations were performed in the pH range of 2.0–11.0 in equilibrium-controlled mode, during which the pH equilibrium was assumed to be reached if a change in the measured potential was less than 0.1 mV within 90 s. The minimum waiting time was 2 min while the maximum was 40 min due to the quite slow equilibrium processes.

The protonation constants of the ligands and the overall stability constants of the complexes, *β*_p,q,r_ = [Pd_p_H_q_L_r_]/[Pd]^p^[H]^q^[L]^r^ (where (“Pd” stands for Pd^2+^, “L” represents the completely deprotonated form of the ligand (L1^−^)) were calculated with the aid of the SUPERQUAD [23] and PSEQUAD [24] computer programs, respectively. The stability constants for the complexes formed in the Pd(II)–Cl^−^ system were taken from the literature (log*β* [PdCl]^+^ = 4.47, log*β*[PdCl_2_] = 7.76, log*β* [PdCl_3_]^−^ = 10.17, log*β*[PdCl_4_]^2−^ = 11.56) [25]. These fixed values were used in the equilibrium models during calculations, but the stability constants for various ternary complexes containing coordinated chloride ions were not assumed. Consequently, the stability constants determined for the various complexes between Pd(II) and the ligands are conditional values that are valid only under the experimental conditions used and the free coordination site(s) of the metal ion is/are occupied by either chloride ion(s) or water molecule(s). “H_–x_” in the formulae of the complexes refers either to the deprotonation of a coordinated water molecule in a Pd(II)–L1^−^ complex or to the metal ion-assisted deprotonation of the very weakly acidic amide group(s) of the bound ligand.

^13^C NMR spectra were recorded on a Bruker AM360, while ^1^H NMR titrations were performed using a Bruker Avance DRX 400 instrument at 25 °C and I_total_ = 0.20 M (0.10 M KCl and 0.20 M KNO_3_). Chemical shifts are reported in ppm (δ_H_) from TSP as an internal reference. The titrations were performed in D_2_O at c_ligand_ = 5 mM using 1:2, 1:1, and 2:1 metal ion to ligand ratios. The pH* of the samples was adjusted with NaOD or DNO_3_. Individual samples with different pH* values were equilibrated for 60 min before measurements. The pH* values (direct pH-meter reading in D_2_O of a pH-meter calibrated in H_2_O according to Irving et al. [22] were converted to pH values using the following equation: pH = 0.936 · pH* + 0.412 [26]. NMR spectroscopy was also used for the study of the protonation and complexation processes of the ligand. As the proton exchange reactions of the ligand were found to be fast in the NMR time scale, the chemical shift of a single resonance in the spectra (δ_obsd._) is the molar fraction weighted average of the chemical shifts belonging to the protonated and deprotonated species (δ_HiL_). The [H^+^] dependence of the chemical shift of a resonance sensitive to the protonation process(es) is given by the following equation:

$$\delta_{\mathrm{obsd.}}=\overset{n}{\underset{i=0}{\sum }}\delta_{H_{i}L}\frac{\beta_{i}{\left[H^{+}\right]}^{i}}{\sum_{j=0}^{n}\beta_{j}{\left[H^{+}\right]}^{j}}$$

where “*n*” is the total number of protonation sites of the ligand, *β* is the overall protonation constant of the ligand and *β*_0_ = 1 by definition [27]. The calculation of p*K* values of H(L1) was carried out via fitting the *δ*_obsd._-pH curves using the Scientist 3.0 computer program and the above equation.

For the study of the complexation processes between [Co(tren)Cl_2_]Cl and H(L1), the solid compounds were dissolved in 12 mL D_2_O (c_Co_ = c_L_ = 12 mM) and one equivalent of NaOD was added. The purple-colored mixture was stirred for 4 h at 60 °C and left to cool down. The resulting solution was used for the study of pH-dependence of the NMR resonances of the [Co(tren)H(L1)]^3+^ and Pd(II)-[Co(tren)H(L1)]^3+^ 1:1 systems in the presence of 0.10 M KCl and 0.10 M KNO_3_ (I_total_ = 0.20 M). The NMR spectra were analyzed using the MestreNova program.

## 3. Results and Discussion

### 3.1. Synthesis and Characterization of the Novel Peptide Conjugate, H(L1)

Synthesis of H(L1) was carried out in a similar manner as for the tripeptide analogue but using an l-Ala-l-Ala building block (Supplementary Materials Figure S1) [5]. Briefly, Bz-protected maltol was reacted with ethylenediamine, and the 1-(2-aminoethyl)-3-benzyloxy-2-methyl-4-pyridinone obtained was further reacted with the Z-protected l-Ala-l-Ala derivative activated at the C terminus with ethylchloroformate in dry THF. Hydrogenolysis in the presence of Pd/C catalyst afforded the final product as a trifluoroacetate salt in moderate yield (see Figure S1). The identity and purity of the ligand were confirmed using various NMR techniques (see Figures S2 and S3), elemental analysis, and pH-potentiometry.

### 3.2. Proton Dissociation Processes of H(L1)

**Figure 1:**
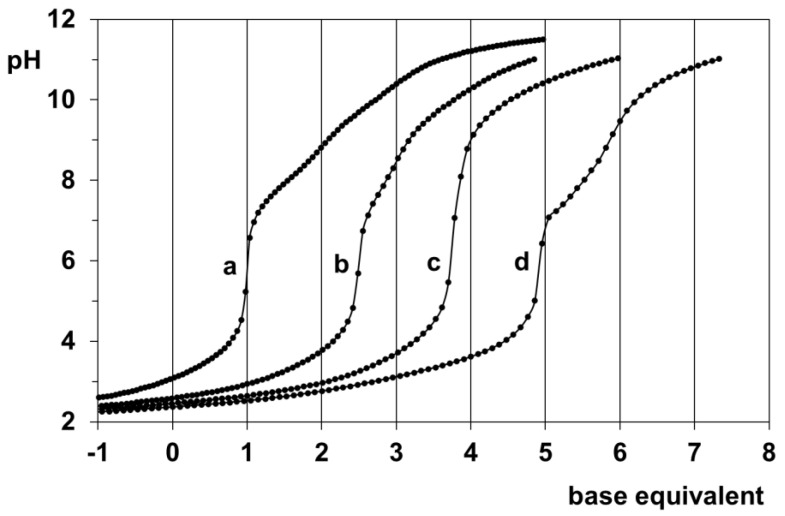
pH-potentiometric titration curves with H(L1) for the H^+^−ligand system (**a**), and the Pd(II) −ligand systems at 1:2 (**b**), 1:1 (**c**), and 2:1 (**d**) ratios. Negative base equivalent refers to an excess of acid in the sample.

Proton dissociation constants of H(L1) were determined with the aid of pH-potentiometric and ^1^H NMR measurements. The fully protonated form of the ligand (see Scheme 1) contains three protons to dissociate. As can be seen in the titration curve of the ligand (Figure 1a), the first deprotonation process takes place in the range 2.5 < pH < 5.0 and belongs to the proton loss of the -OH(4) group of the pyridinone ring followed by the deprotonation of the ammonium and pyridinone -OH(3) groups in overlapping processes above pH 7. This is also supported by the NMR titration data of H(L1) (see Figure S4). Figure S4 reveals that the resonance of the Ala methyl protons in the vicinity of the ammonium group exhibits a shift in the range 7.0 < pH < 9.0 (consistent with the p*K* = 7.9 value), while for the pyridinone ring H two processes can be seen on increasing the pH (Figure S4). We have previously studied in detail the -NH vs. -OH deprotonation at the pyridinone ring [28] concluding that the deprotonation of the model H(L4) molecule occurs mostly at the -OH rather than the -NH part of the ligand (regarding the lower p*K* = 3.2 value) and the higher p*K* = 9.6 value belongs to the other -OH group. Due to the ambidentate character of H(L1), the same (de)protonation sequence and processes are assumed for the pyridinone ring here too. The obtained dissociation constants are summarized in Table 1. For comparison, p*K* values of the tripeptide-HP analogue (H(L2), see Scheme 1), l-alanyl-l-alanine (H(L3)), and 3-hydroxy-1,2-dimethylpyridin-4(1H)-one (H(L4)) are also summarized in Table 1. Analysis of the data for H(L1) in Table 1 shows that the pH-potentiometric and ^1^H NMR results are in good agreement. p*K*_a_ values of the dipeptide conjugate are very similar to those of H(L2) while deprotonation of the corresponding functions occurs at a pH lower by 0.2–0.5 unit compared to that of H(L3) and H(L4).

### 3.3. Pd(II) Complexation of H(L1)

pH-potentiometric study of the Pd(II)-H(L1) system at various metal ion to ligand ratios showed that the complex formation processes are slow in this system too, similarly to previous systems where a peptide backbone is available for coordination [5,7,20]. Nevertheless, the titration curves in Figure 1 indicate that by pH~5 the ligand is capable of binding two Pd(II) ions based on the five equivalent of base consumption at a 2:1 metal ion to ligand ratio. At a 1:1 metal ion to ligand ratio, the not exactly four equivalents of base consumption may refer to the not complete equilibrium state of the sample or the co-presence of various species with different stoichiometry. The lack of extra base consumption for the 1:2 sample is in line with the absence of any 1:2 species in a measurable concentration.

**Figure 2:**
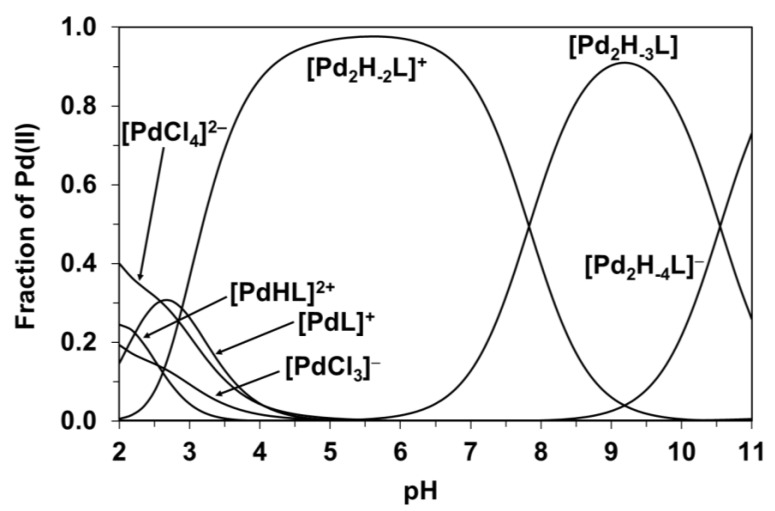
Calculated concentration distribution curves for the Pd(II)−(L1)^−^ system at a 2:1 ratio, c_L_ = 5 mM. “L” refers to the L1 ligand in the formulae of the various species.

Evaluation of the titration curves resulted in the model and stability constants presented in Table 2. For comparative purposes, Table 2 also shows the appropriate data previously obtained for the Pd(II)-(L2)^−^ system (see Scheme 1) under identical conditions [5]. The speciation diagram for the Pd(II)-(L1)^−^ system is presented in Figure 2. Since the log*β* values in Table 2 can only be considered as tentative, detailed NMR measurements were also carried out to support this model and to explore the most likely solution structure of the major species formed.

As Figure 2 indicates, at a 2:1 metal ion to ligand ratio, the complexation starts with the formation of [PdHL]^2+^, but in strongly overlapping processes, [PdL]^+^ is also present. On increasing the pH further in the 2:1 system, [Pd_2_H_–x_L] type species as major complexes predominate until pH = 11.

**Figure 3:**
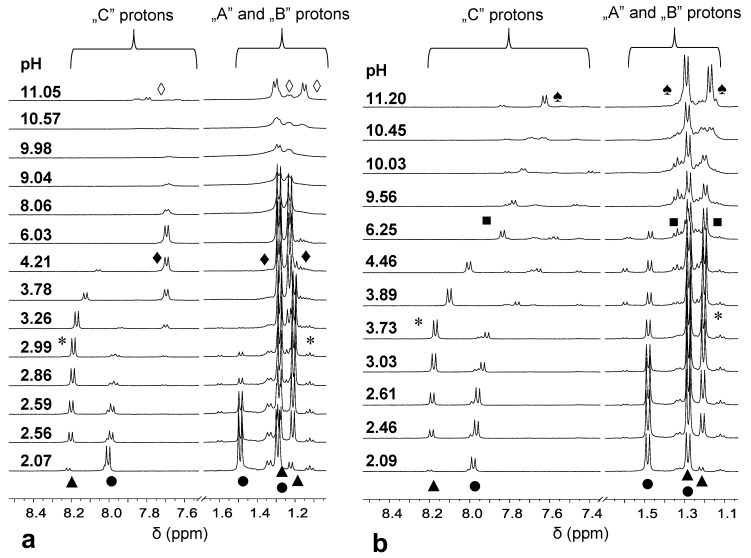
pH-dependence of the NMR signals belonging to the Ala -CH_3_ protons (A and B) and pyridinone -CH protons (C) registered at 2:1 (**a**) and 1:1 (**b**) Pd(II) to ligand ratio with L1. For notations, see Scheme 1. Symbols represent the signals belonging to the following species: [H_3_(L1)]^2+^ (●), [PdHL]^2+^ (▲), [PdL]^+^ (*), [Pd_2_H_–2_L]^+^ (♦), [Pd_2_H_–3_L)] and [Pd_2_H_–4_L)]^−^ (◊), [PdH_–1_L] (■), [PdH_–3_L]^2−^ (♠).

NMR spectra of the 2:1 system acquired at different pH values (Figure 3a) provided support for the speciation. As seen in Figure 3a, at pH = 2.07, besides the resonances of the free H_3_(L1)^2+^ ligand (●), new signals (▲) both for the “C” proton of the pyridinone ring and for the methyl doublets of the dipeptide backbone (“A” and “B” protons) appear. The large upfield shift of the “A” and “B” protons in agreement with earlier findings with the tripeptide conjugate system [5] strongly suggests the formation of an (NH_2_,N_amide_) coordinated complex (see the suggested structures of the various species in Figure S5). Previous studies have also shown a large upfield shift of the DHP ring protons upon (O,O) coordination of the model pyridinone (H(L4)) ligand to Pd(II). The lack of the upfield shift in the signal belonging to the “C” proton in Figure 3a, thus, also supports the (N,N) binding mode of H(L1) in the complex formed at pH 2.07, while the DHP entity is non-coordinated. Since the pyridinone moiety within the H(L1) ligand is still doubly protonated, the complex assigned by (▲) should have a [PdHL]^2+^ stoichiometry in agreement with the pH-potentiometric results.

On increasing the pH, by pH ~2.8, the (▲) signals of the “A” and “B” protons exhibit a slight upfield shift, which is consistent with the formation of [PdL]^+^ (*) in agreement with the speciation data in Figure 2. [PdL]^+^ has most likely an (NH_2_,N_amide_,N_amide_) binding mode and it is in fast exchange with [PdHL]^2+^ (▲) on the NMR time scale. The 3xN coordination mode in [PdL]^+^, rather than the additional participation of the DHP O-donors in Pd(II) binding, is also supported by the significant upfield shift of the “C” proton of the non-coordinated DHP ring only above pH 3.3 due to deprotonation at this site. The appearance of new signals (♦) in the range 3 < pH < 8 accompanied by a large upfield shift of the “C” protons (but an almost negligible shift of the “A” and “B” protons) is consistent with the binding of another Pd(II) ion resulting in the formation of [Pd_2_H_–2_L]^+^ with (O,O) and (NH_2_,N_amide_,N_amide_) coordinated metal ions. Above pH ~ 8, the significant broadening of the signals suggests fast exchange processes in the system, while upon increasing the pH further, a new species (◊) becomes predominant. By also taking into consideration the speciation curves (Figure 2), these findings can be rationalized by the partial hydrolysis of the two Pd(II) ions coordinated via (O,O) and (NH_2_,N_amide_,N_amide_) manner, respectively, resulting in the formation of mixed hydroxido complexes where the free coordination sites of the metal ions are taken by hydroxide ion(s).

The pH dependent NMR spectra registered at a 1:1 ratio and below pH ~3 reveal similar complexation processes as at a 2:1 ratio with the presence of [PdHL]^2+^ (▲) and [PdL]^+^ (*) with (NH_2_,N_amide_) and (NH_2_,N_amide_,N_amide_) binding modes, respectively (Figure 3b). Above pH ~ 3.8, a continuous upfield shift of the signals of the “C” proton is consistent with the deprotonation of the hydroxyl group in position 4 of the DHP unit. The [PdH_–1_L] (■) formed in this way is a major species over a wide pH range. The further upfield shift of the “C” signal above pH ~9 is in line with the second proton loss of the non-coordinated DHP ring. Comparable values of p*K*_PdH__-1L_ (Table 2) and p*K*_OH(3)_ (Table 1) provide further support for the deprotonation of the non-coordinated -OH function of the DHP entity. At pH > 10, a slightly broadened set of new signals (♠) is consistent with the formation of a mixed hydroxido complex, [PdH_–3_L]^2−^, also detected by pH-potentiometry.

A comparison of the Pd(II) binding strengths of the tripeptide and dipeptide HP conjugates reveals that the peptide backbone is the major metal ion binding site in both cases. At the same time, a detailed analysis of the various derived p*K* values (Table 2) together with the results of NMR studies shows that for H(L1), the metal ion-assisted deprotonation and coordination of the second amide group via the N atom occurs at lower pH than for the tripeptide conjugate. This difference can be rationalized by the fact that for H(L2), the second amide group puts together two amino acid moieties, while for the H(L1) derivative, the second amide is connected directly to the ethylene chain attached to the ring N of the DHP unit. This results in slightly higher acidity of the amide function in the latter case, allowing metal ion-assisted deprotonation at a lower pH. The higher stability of the (NH_2_, N_amide_, N_amide_) binding mode with H(L1) may also explain why no dimeric complexes with [Pd_2_H_–1_L]^2+^ composition are formed, unlike in the Pd(II)−H(L2) system.

### 3.4. Formation of Heterobimetallic Complexes with H(L1) in Solution

Donor atom preference of [Co(tren)]^3+^ and Pd(II) towards the ambidentate H(L1) was studied in order to explore the formation of exclusive binding modes, allowing the development of hypoxia-activated heterobimetallic complexes.

**Figure 4:**
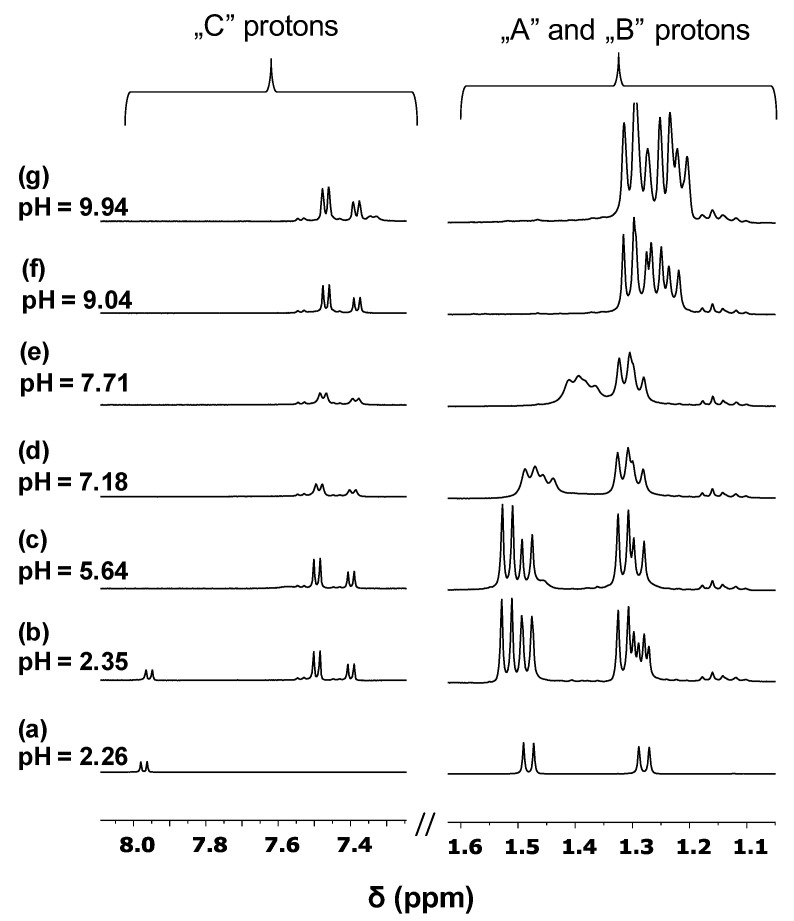
^1^H NMR spectra registered for the H^+^–(L1)^−^ (**a**) and [Co(tren)]^3+^–(L1)^−^ system (**b**–**g**) at different pH values.

Complexation of [Co(tren)Cl_2_]Cl with H(L1) in the presence of one equivalent base was studied at different pH values by ^1^H NMR. The obtained spectra are shown in Figure 4b–g together with the spectrum of the free ligand (a). A comparison of Figure 4a and Figure 4b reveals that at pH 2.35, besides some free ligands, new signals belonging to the complex are present. As it can be seen in Figure 4b, complex formation starts even under strongly acidic conditions; signals of -CH proton belonging to the pyridinone ring (“C”) show a larger upfield shift, while for Ala-CH_3_ protons (“A” and “B”), only a slight downfield shift can be observed compared to the free ligand. This clearly supports the (O,O) coordination of the ligand to [Co(tren)]^3+^. On increasing the pH, the pyridinone part remains coordinated in the whole pH range studied, while the significant upfield shift in the aliphatic region in the range 7 < pH < 9 can be attributed to the deprotonation of the ammonium group. “A” protons—due to their vicinity to the ammonium function—are more sensitive to deprotonation than “B”, leading to a larger upfield shift for the former one. The pH range of the proton loss is very similar to that found for the free ligand (see Table 1), supporting the peptide backbone to remain uncoordinated. In addition, duplication of the signals in Figure 4b–g indicates the presence of binding isomers due to the asymmetric nature of the (O,O) chelate coordinated to the octahedral Co(III) entity. The roughly 2:1 isomer ratio remains unchanged in the whole pH range suggesting the formation of major and minor species. These findings provide solid evidence for the exclusive coordination of [Co(tren)]^3+^ to the pyridinone part of the ligand resulting in the formation of a stable and inert complex over a wide pH range.

**Figure 5:**
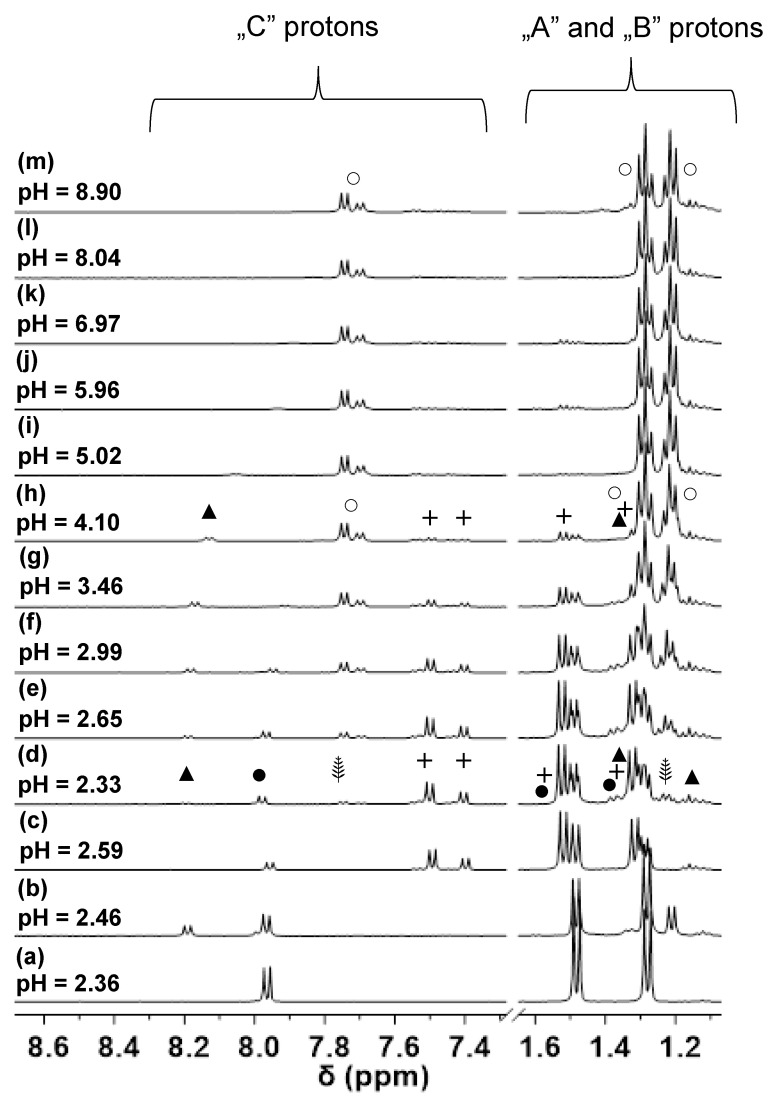
^1^H NMR spectra registered for the H^+^–(L1)^−^ (**a**), the Pd(II)–(L1)^−^ (**b**), or [Co(tren)]^3+^–(L1)^−^ (**c**) monometallic and the Pd(II)–[Co(tren)]^3+^–(L1)^−^ (**d**–**m**) bimetallic systems at different pH values. Symbols represent the signals belonging to the following species: [H_3_(L1)]^2+^ (●), [PdHL]^2+^ (▲), [Co(tren)]^3+^ (+), [PdH_–1_LCo(tren)]^3+^ (⸙), [PdH_–2_LCo(tren)]^2+^ (○).

^1^H NMR spectra obtained for the samples containing both [Co(tren)]^3+^ and Pd(II) metal ions are presented in Figure 5. As it can be seen in Figure 5d, besides the presence of the free ligand (●), the Co(III) complex (+) and [PdHL]^2+^ (▲), two new sets of doublets (⸙) appear in the reaction mixture. Analysis of the integral values revealed a 2:1 ratio for the (⸙) signals suggesting, therefore, the formation of further [Co(tren)]^3+^ containing complexes. The intensity of these new signals increases with increasing the pH; furthermore, a continuous downfield shift of Ala-CH_3_ protons (A and B) can be detected in the range 2.33 < pH < 2.99. These findings indicate the formation of two complexes being in fast ligand exchange with each other on the NMR time scale. Taking into consideration that the pH range for the formation of [PdHL]^2+^ and [PdL]^+^ was found to be very similar and a fast exchange was also observed in this system (▲ and * in Figure 3, respectively), it is plausible to assume the formation of Co(III)-Pd(II) bimetallic complexes.

In these dinuclear species, (L1)^–^ coordinates to [Co(tren)]^3+^ through an asymmetric (O,O) chelate yielding isomers with a roughly 2:1 ratio while coordination to Pd(II) occurs via (NH_2_,N_amide_) and (NH_2_,N_amide,_ N_amide_) chelates in (⸙) and (○), respectively. The latter donor set provides a high stability resulting in (○) being the single complex over a wide pH range. Based on the obtained results, H(L1)—similar to the tripeptide derivative, H(L2), [5]—is also a suitable ligand for designing heterobimetallic Co/Pd(Pt) complexes for selective activation under hypoxic conditions.

## 4. Conclusions

Multitargeted, bimetallic complexes incorporating an inert Co(III) chaperon entity and a platinum group metal with antiproliferative activity may be promising novel candidates to overcome the limited selectivity of the platinum(II) drugs currently used in chemotherapy. In this study, the synthesis and characterization of a novel dipeptide conjugate, H(L1), are described. For the successful synthesis of the mentioned heterobimetallic complexes, and to explore their solution behavior of them after administration, it is also desirable to explore the metal ion preference and the strength of metal binding of the donor atom sets of the novel H(L1). For this purpose, to model the rather inert Pt(II), the complexation with Pd(II) was studied using pH-potentiometric and NMR techniques. Notably, numerous Pd(II) complexes themselves exhibit anticancer potential.

Our results show that at a 1:1 metal ion to ligand ratio, the expected coordination of Pd(II) to the peptide backbone of the ligand occurs, resulting in the formation of an N-coordinated species with the involvement of an increasing number of peptide nitrogens besides the anchoring terminal amino group on increasing the pH. H(L1), however, is also capable of binding two Pd(II) in solution forming [Pd_2_H_–n_(L1)] (n = 1–3) complexes via the coordination both of the (O,O) and (N,N) chelating sets over a wide pH range. Comparison of the Pd(II) binding strengths of the novel di- and the tripeptide conjugates [5] revealed the slightly higher effectivity of the former one due to higher acidity of the second amide group in the vicinity of the linker alkyl group. An NMR study has also revealed that the reaction of H(L1) with [Co(tren)]^3+^ resulted in the exclusive formation of the (O,O) coordinated Co(III) complex, and upon addition of Pd(II), the Co/Pd complex with the expected coordination mode was formed in solution.

All these results pave the way for the desired solid heterobimetallic complexes and this work is currently in progress in our laboratory.

## Figures and Tables

**Table 1:** Proton dissociation constants (p*K*_a_) of H(L1) and some model ligands at t = 25.0 °C and I_tot_ = 0.20 M (0.10 M KCl + 0.10 M KNO_3_) *.

	H(L1)		H(L2) [5]	H(L3) [29]	H(L4) [28]
	pH-Potentiometry	^1^H NMR	pH-Potentiometry		
p*K*_COOH_	-	-	-	3.08	-
p*K*_OH(4)_	3.22(3)	3.46(5)	3.32	-	3.70
p*K*_NH3_^+^	7.88(2)	7.70(2)	7.74	8.26	-
p*K*_OH(3)_	9.64(1)	9.56(4)	9.46	-	9.76

* 3σ standard deviations are parentheses.

**Table 2:** Overall stability constants (log*β*) of the Pd(II) complexes formed with H(L1) and H(L2) at 25.0 °C and I_tot_ = 0.20 M (0.10 M KCl + 0.10 M KNO_3_) *.

	H(L1)	H(L2) [5]
[PdHL]^2+^	26.84(1)	28.18
[PdL]^+^	24.62(1)	24.54
[PdH_–1_L]	20.35(3)	21.01
[PdH_–2_L]^−^	10.39(5)	12.7
[PdH_–3_L]^2^^−^	−0.71(5)	2.4
[Pd_2_H_–1_L]^2+^	-	32.71
[Pd_2_H_–2_L]^+^	28.66(2)	28.99
[Pd_2_H_–3_L]	20.82(4)	21.8
[Pd_2_H_–4_L]^−^	10.27(5)	11.8
p*K*_PdLH_	2.22	3.64
p*K*_PdL_	4.27	3.53
p*K*_PdH__-1L_	9.96	8.31
p*K*_PdH__-2L_	11.10	10.30
p*K*_Pd__2H__-1L_	-	3.72
p*K*_Pd__2H__-2L_	7.84	7.20
p*K*_Pd__2H__-3L_	10.55	10.0

* 3σ standard deviations are in parentheses.

## Data Availability

Not applicable.

## Supplementary Materials

The following supporting information can be downloaded at: https://www.mdpi.com/article/10.3390/molecules27144667/s1, Figure S1: Synthetic route for H(L1).; Figure S2: ^1^H NMR spectrum of H(L1)·CF_3_COOH, registered in D_2_O; Figure S3: ^13^C NMR spectrum of H(L1)·CF_3_COOH, registered in D_2_O; Figure S4: pH-dependence of the ^1^H NMR signals belonging the Ala methyl (red) and pyridinone ring hydrogen (blue).; Figure S5: Suggested solution structures of the various complexes.
