# Supplementary material for: Pd(II) Binding Strength of a Novel Ambidentate Dipeptide-Hydroxypyridinonate Ligand: A Solution Equilibrium Study
Source: Molecules. 2022 Jul 21;27(14):4667. doi: 10.3390/molecules27144667 (PMC9324670; doi:10.3390/molecules27144667)
Supplement: Supplementary file 1 [file molecules-27-04667-s001.zip › molecules-1713748-supplementary.pdf]

Pd(II) binding strength of a novel ambidentate dipeptide-hydroxypyridinonate ligand; a solution equilibrium study

Linda Bíró, András Ozsváth, Réka Kapitány, Péter Buglyó *

Department of Inorganic & Analytical Chemistry, Faculty of Science and Technology, University of Debrecen, H-4032 Debrecen, Egyetem tér 1, Hungary

Supporting Information

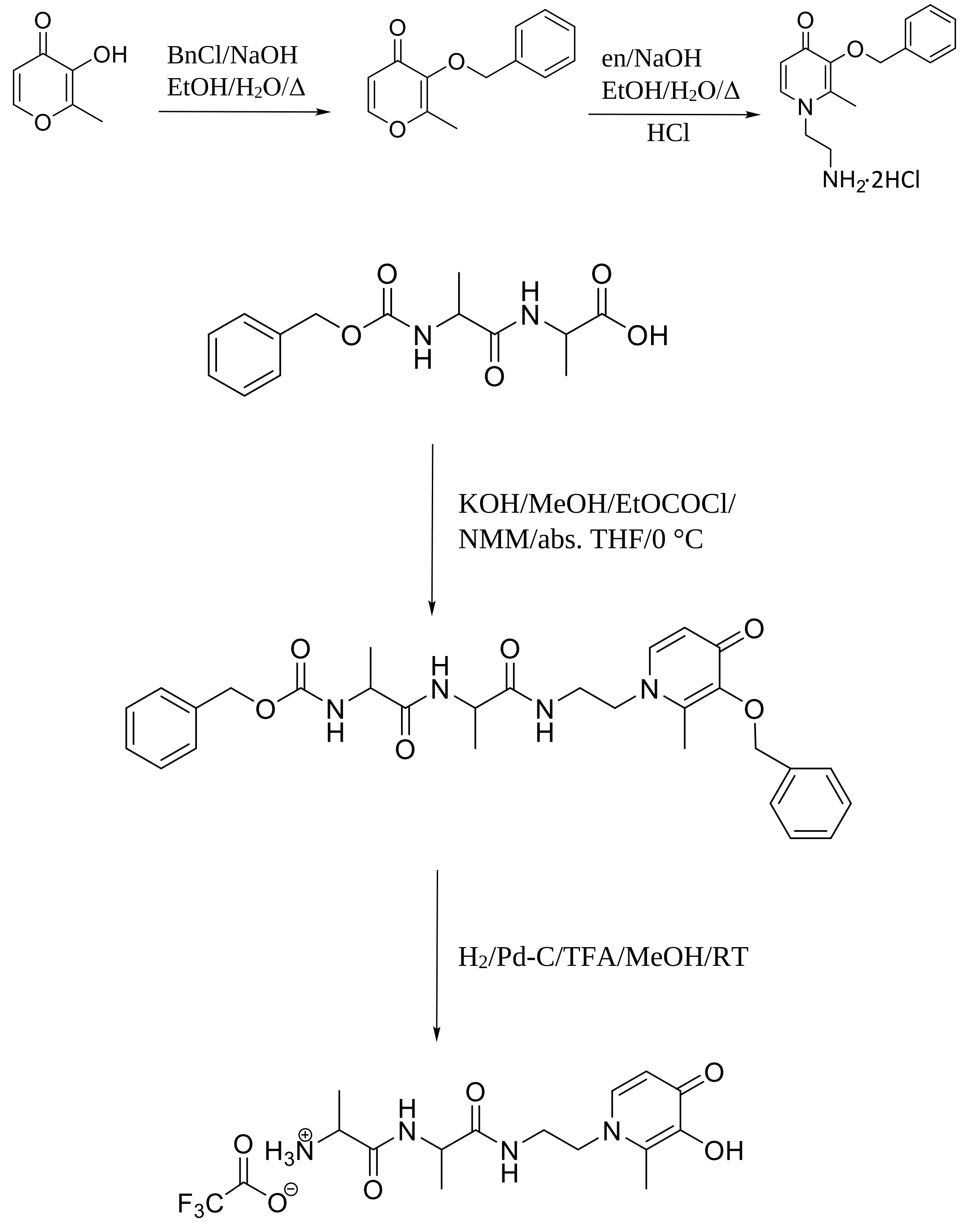

Figure S1. Synthetic route for H(L1).

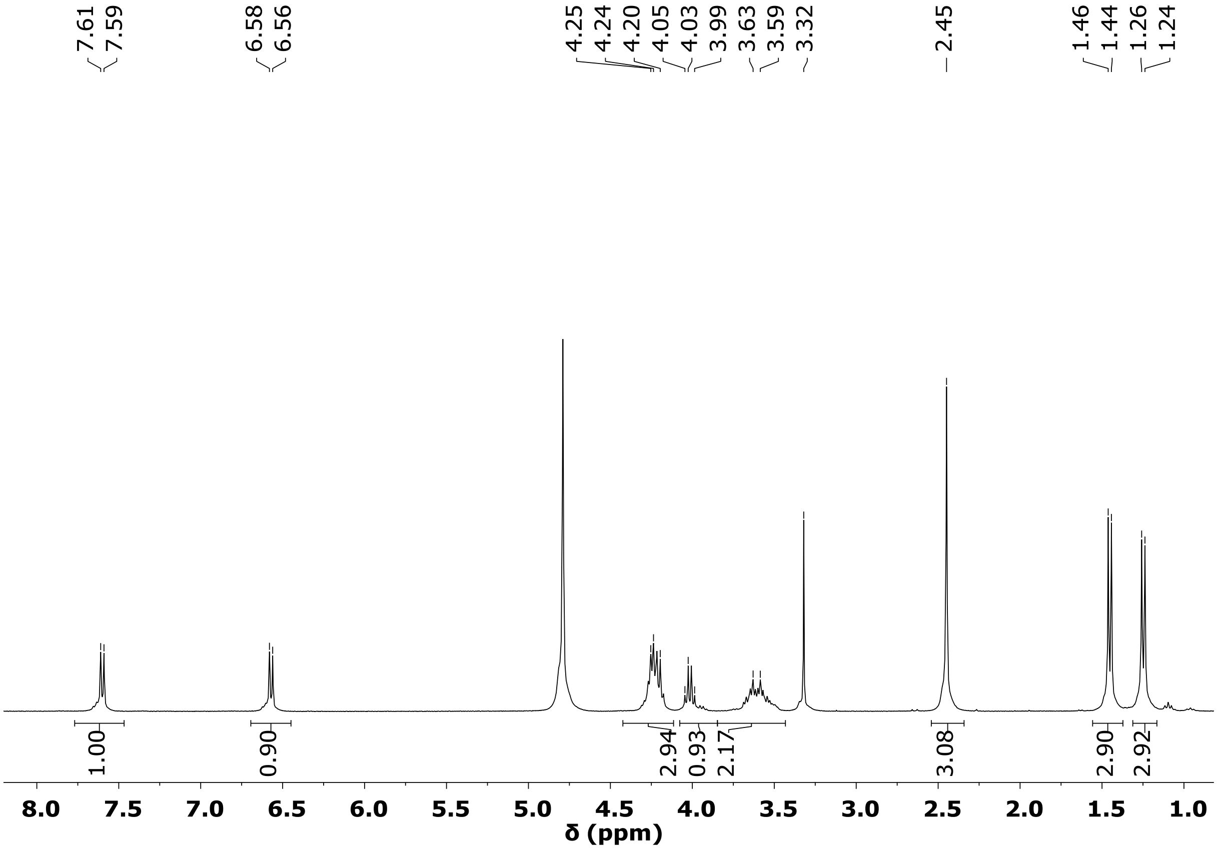

Figure S2. ^1H NMR spectrum of H(L1)·CF_3COOH, registered in D_2O. The signal at 3.34 ppm corresponds to methanol.

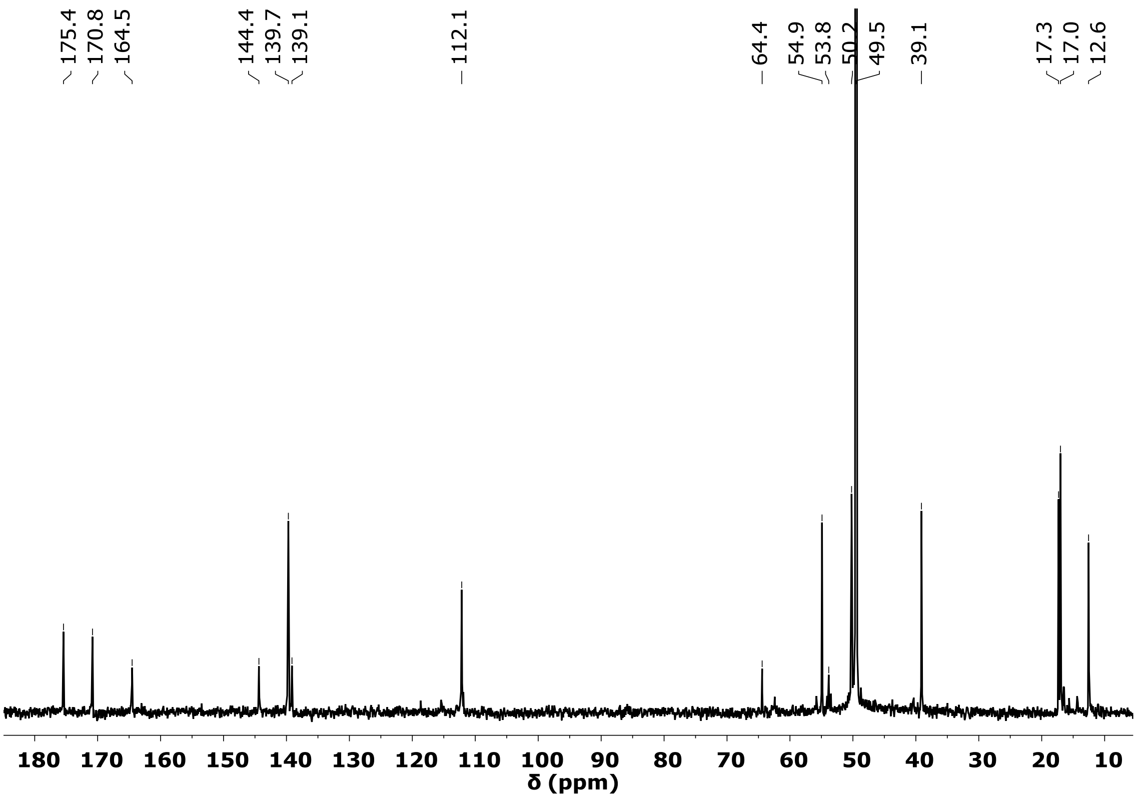

Figure S3. ^{13}C NMR spectrum of H(L1)·CF_3COOH, registered in D_2O. Calibration was done using the methanol signal at 49.5 ppm.

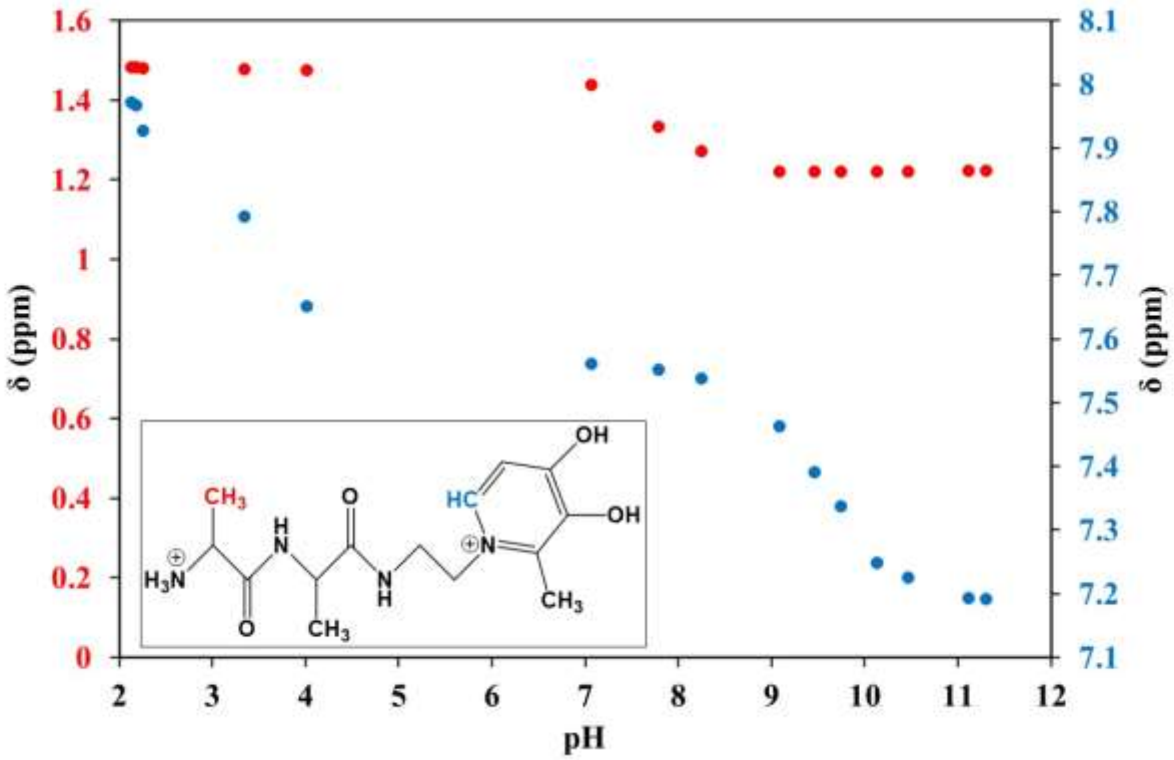

Figure S4. pH-dependence of the ^{1}H NMR signals belonging the Ala methyl (red) and pyridinone ring hydrogen (blue).

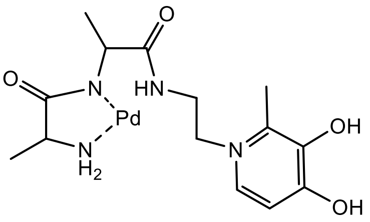

[PdHL]

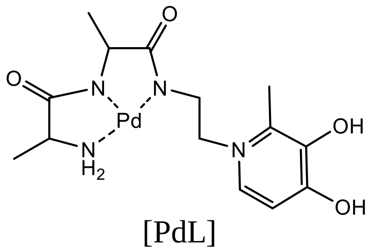

[PdL]

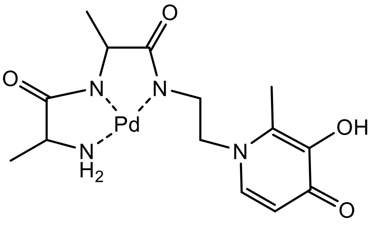

$[PdH_{-1}L]$

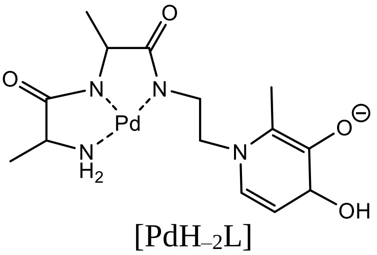

$[PdH_{-2}L]$

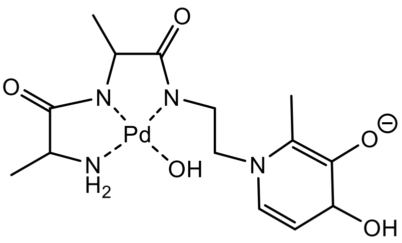

$[PdH_{-3}L]$

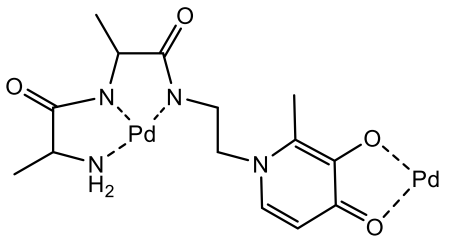

$[Pd_2H_{-2}L]$

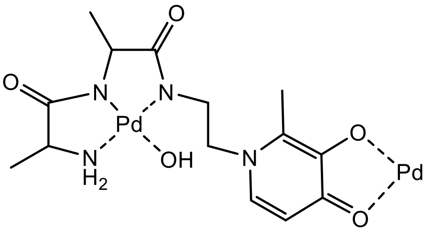

$[Pd_2H_{-3}L]$

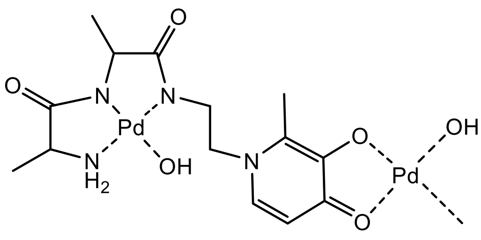

$[Pd_2H_{-4}L]$

Figure S5. Suggested solution structures of the various complexes. The vacant coordination sites of the Pd(II) ions are taken either by water molecules or chloride ions therefore the overall charges of the complexes are omitted.
